# Career preferences of graduating medical students in China: a nationwide cross-sectional study

**DOI:** 10.1186/s12909-016-0658-5

**Published:** 2016-05-06

**Authors:** Jianlin Hou, Maoyi Xu, Joseph C. Kolars, Zhe Dong, Weimin Wang, Amy Huang, Yang Ke

**Affiliations:** Institute of Medical Education, Peking University, Beijing, China; China Medical Tribune, Beijing, China; University of Michigan Medical School, Ann Arbor, MI USA; Peking University Health Science Center, Beijing, 100191 China

**Keywords:** Career preference, Graduating medical students, Rural health workforce, Primary care provider, China

## Abstract

**Background:**

China faces major challenges in the distribution of health professionals with serious shortages in rural areas and in the development of Primary Care Providers (PCPs). This study investigates the career preferences of medical students in China and the impact of rural backgrounds on these preferences.

**Methods:**

Medical students in the final year of their program in 16 medical schools across China completed a 58-item survey that included questions regarding their demographic characteristics, attitudes toward practice in low resource areas, postgraduate planning, self-assessed competency, university facilities assessment, and financial situation. Descriptive calculation and Logit model were used for the analysis.

**Results:**

Completed surveys from 3020 students were included in the analysis. Upon graduation, 48.5 % of the medical students preferred to work in urban public hospitals and this percentage rose to 73.6 % when students were asked to state their anticipated preference five years after graduation. Students’ top three ranked reasons for preferred careers were “good career prospects”, “living close to parents/families”, and “remuneration”. Those who preferred to work in rural areas upon graduation were more likely to be those who lived in rural areas when 1–15 years old (β = 2.05, *p* < 0.001), had high school in rural areas (β = 1.73, *p* < 0.001), or had parents’ place of current residence in rural areas (β = 2.12, *p* < 0.001). Similar results were found for those students who preferred to work in PCPs.

**Conclusions:**

To address the serious shortages of health professionals in rural areas and PCPs, medical schools should consider strategies to recruit more medical applicants with rural backgrounds and to orient students to rural and primary care interests.

**Electronic supplementary material:**

The online version of this article (doi:10.1186/s12909-016-0658-5) contains supplementary material, which is available to authorized users.

## Background

China faces major challenges in the distribution of health professionals throughout the country. Urban hospitals attract most of the health professionals with serious shortages in rural areas where approximately 50 % (642 million) of the population currently resides. In 2012, the following gaps were observed between rural and urban areas for health professionals (3.41 VS 8.55), doctors (1.00 VS 2.96), registered nurses (RNs) (1.09 VS 3.65) per 1000 population [1]. Compared to physicians in urban areas, doctors in rural areas were characterized as having lower levels of educational level, professional knowledge, and competency [2, 3]. In township health centers, which serve as the focus of care in rural settings, less than 50 % of physicians and physician assistants had an educational level beyond secondary school. The inadequacy in the number and professional capabilities of health professionals in rural areas contributes to decreased access to health care for rural residents, relative to their urban counterparts [4].

Urban hospitals often define themselves as referral centers that focus on secondary or tertiary care. Primary health care has been relatively under-developed but more recently identified as one of three key principles for healthcare reform in China, because it plays an increasingly important role in promoting population health, addressing chronic diseases, and reducing health expenditures [5, 6]. In order to guarantee the availability of primary health care, properly trained health professionals must be appropriately distributed in order to serve the entire population. Nevertheless, reports across China consistently show that shortage of health professionals is one of the most frequently cited issues that threatens the survival and development of primary care providers (PCPs) [7–11].

Despite problems regarding the distribution of health professionals to rural areas and the need for expertise in primary care, the number of newly employed health workers accounted for only 20–40 % of graduates between 2004 to 2008 [12]. Many graduates seek employment in fields outside of healthcare [13]. This indicates an inefficiency of the governments’ educational investments to increase the healthcare workforce which, if corrected, could potentially enhance the distribution of healthcare professionals.

Similarly, few medical students choose to focus their careers in underserved areas or in primary care [14, 15]. It was reported in one survey that about 73 % of students (*n* = 700) in a 3-year medical curriculum preferred to work in urban big-sized hospitals [16]. Data from another medical school shows that the percentage of graduates entering primary care was noted to be decreasing (i.e. 26 % in 2009, 23 % in 2010, and 21 % in 2011) [17].

To address the lack of interest in primary care or in rural practice, it is important to understand the career preferences of medical students and variables that influence these choices. Research to date has been limited because of small sample size, convenience sampling, confounding factors, and single institution studies [18–21].

The purpose of this study is to understand the career preferences of a multi-institutional sampling of medical students in China. We hypothesize that medical students who come from rural backgrounds are more likely to work in rural areas and pursue careers in primary care practice than those from more urban backgrounds.

## Methods

### Participants and procedures

Undergraduate medical students at 16 medical schools across China are the focus of this study. Thirteen of the sample medical schools were selected by stratified sampling from the 180 undergraduate medical programs in China. Stratification considered location, ownership (public or private), and designation as a Project 211 university (i.e. one of the 112 key universities selected by the Ministry of Education to receive prioritized funding for a series of education reforms since 1995 [22]). Two leaders with expertise in medical education reviewed the sample cohort and a suggestion was made to add another three leading universities (also Project 211 universities) bringing the final number of sample schools to 16. Four of the sample schools are owned by Project 211 universities. Five, eight, and three of them are located in eastern, middle, and western China, respectively. At each of these medical schools, hard copies of the questionnaire were distributed to all students in their final year of the medical program (year 2012) and collected anonymously, most typically at the end of one of their mandatory lectures.

### Ethical considerations

The study was granted an exemption from requiring ethics approval by Peking University Institutional Review Board because the survey was anonymous and did not include sensitive questions. An introduction about the survey was provided on the first page of the questionnaire, including aims and main contents of the survey, promise to keep the data anonymous and confidential. Participation in the study was voluntary and consent was sought from all participants.

### Questionnaire

The tool used in this study is a self-administered questionnaire that was originally developed in a 5-country project aiming to conduct a situation analysis of health professional education in participating countries. The questionnaire was translated into Chinese and modified for this study in order to better reflect the situation in China.

The modified questionnaire asked information regarding the following seven aspects: demographic characteristics, attitudes toward practice in low resource areas, postgraduate planning, self-assessed competency, university facilities assessment, and financial situation of students and their families. It consisted of 15 fixed response questions, four open-ended items, 39 five-point Likert scale items. The modified questionnaire was pre-tested in a pilot study with students at Peking University Health Science Center.

### Analysis

Descriptive analyses of students’ responses include calculation of percentages, means, and standard deviations. Chi-square tests were conducted to compare participants’ preferences regarding working in rural areas/PCPs by rural background: whether lived in rural areas when 1–15 years old, whether graduated from a high school in rural areas, or whether his/her parents’ place of residence was in rural areas.

Furthermore, Logit models were estimated using Maximum Likelihood Estimation (MLE) techniques to explore the factors predicting students’ preferences regarding working in rural areas/PCPs. The Logit model was defined as follows:

1 $$ \begin{array}{l} \Pr \left({y}_{{}_i}=0\right)=\frac{1}{1+ \exp \left({X}_i\beta +{\mu}_i\right)}\\ {} \Pr \left({y}_i=1\right)=\frac{ \exp \left({X}_i\beta +{\mu}_i\right)}{1+ \exp \left({X}_i\beta +{\mu}_i\right)}\end{array} $$

where *X* was the vector of explanatory variables, *μ*_*i*_ were unobserved group effects, and the *β* were parameters to be estimated.

#### Dependent variables

Dependent variables in the Logit model were students’ preference regarding working in rural areas/PCPs upon graduation (‘yes’ or ‘no’). Rural areas were defined as being located in a county, town, or village [23].

#### Independent variables

For each of the dependent variables, three key independent variables were entered into the model, namely whether students lived in rural areas when 1–15 years old, whether students’ high school was located in a rural area, and whether his/her parents’ place of residence was in a rural area. Other controlling independent variables included age, sex, father’s education, mother’s education, family income per capita per year in the past five years, type of medical school, and region.

All analyses were conducted using StataMP11.0. P value was set at 0.05 to indicate significant differences.

## Results

### Descriptive statistics

Altogether 3052 responses were received, of which 32 ones with missing values were excluded from analysis. As noted in Table 1, the participants’ mean age was 24 years. The family income per capita per year in the past five years was 26718.4 Yuan (about US$ 4312.3). More than half of the participants were female and less than 7 % were from a Project 211 University.

**Table 1:** Summary statistics for graduating medical undergraduates in the study (*N* = 3020)

Variable	Percent or Mean (SD)
Age	23.9(1.1)
Family income (Yuan) per capita per year	26718.4(80979.5)
Sex	
Male	44.1 %
Female	55.9 %
Type of medical school	
Project 211 university	6.5 %
Non-Project 211 university	93.5 %
Region	
Eastern China	30.1 %
Middle China	66.1 %
Western China	3.8 %
Father’s education	
Never attended school	1.0 %
Primary school	9.7 %
High school	48.2 %
Secondary school	10.1 %
Bachelor or diploma	27.1 %
Master	1.8 %
Doctor	1.1 %
Other	1.2 %
Mother’s education	
Never attended school	3.4 %
Primary school	17.6 %
High school	45.5 %
Secondary school	10.4 %
Bachelor or diploma	19.9 %
Master	1.4 %
Doctor	0.8 %
Other	0.8 %
Majority of residence in first years	
Town or village	49.2 %
County	25.4 %
City	24.9 %
Other	0.5 %
Location of high school	
Town or village	10.3 %
County	58.6 %
City	30.2 %
Other	0.9 %
Parents’ place of residence	
Town or village	43.6 %
County	27.3 %
City	28.2 %
Other	1.0 %

Almost half of the respondents’ mother or father had an educational level limited to high school. A substantial proportion of the students lived in towns or villages when 1 to 15 years old, had high school located in counties, and had parents living in towns or villages.

Almost half of respondents (48.5 %) preferred to work in urban public hospitals upon graduation. This proportion rose to 73.6 % when students were asked to state their anticipated preference five years after graduation. Other chosen preferences upon graduation were pursuing graduate study (37.6 %), public primary care provider (8.1 %) and private hospital/clinic (2.9 %). The remaining preferences upon graduation accounted for less than 1 %, including academic employment, going abroad, being self-employed, medical or pharmaceutical company, and non-governmental organization (Table 2). Male respondents’ career preferences were different from female ones, such as public hospital (53.1 % VS 44.8 %), pursuing graduate study (33.1 % VS 41.1 %) (Additional file 1). Furthermore, 71.8 % of the medical undergraduates preferred to work in cities. In contrast, only 23.8 % preferred to work in counties and 2.7 % preferred to work in towns or villages.

**Table 2:** Graduating medical undergraduates’ career preference (%)

Preferred career	Upon graduation (*n* = 3020)	Five years after graduation (*n* = 3020)
Public hospital	48.5	73.6
Pursuing graduate study	37.6	8.1
Public primary care provider	8.1	7.0
Private hospital/clinic	2.9	2.1
Academic employment	0.7	2.2
Going abroad	0.6	2.5
Self-employed	0.4	1.9
Medical/pharmaceutical company	0.3	0.6
Non-governmental organization	0.3	0.4
Other	0.8	1.7
Total	100.0	100.0

As shown in Table 3, the top three ranked reasons for preferred careers were “good career prospects”, “living close to parents/families”, and “remuneration”. Furthermore, the top ranked reason by male and female respondents was “remuneration” and “good career prospects”, respectively (Additional file 2).

**Table 3:** Reasons for preferred career (%)

Reasons	1^st^ reason (*n* = 2,706)	2^nd^ reason (*n* = 2,685)	3^rd^ reason (*n* = 2,678)
Good career prospects	29.1	17.0	15.3
Living close to parents/families	26.9	17.7	11.5
Remuneration	20.8	20.6	18.5
Nice place to live	5.8	10.7	11.3
Return to hometown	4.6	7.2	5.2
Social prestige	3.2	4.9	7.8
Work environment	3.1	7.1	11.9
Opportunity for further training	2.9	7.2	6.6
Get away from parents	2.3	3.0	1.3
Good welfare	0.9	3.5	7.5
Housing	0.3	0.9	2.7
Other	0.4	0.2	0.3
Total	100.0	100.0	100.0

### Inferential statistics

According to results from Chi-square tests (Table 4), it seems that there is a relationship between preference for working in rural areas/PCPs and rural background.

**Table 4:** Preference for rural areas and PCPs by past experiences in rural areas

	Preference for practice in rural areas upon graduation		Chi-square	Preference for public PCP upon graduation		Chi-square
	Yes *n* = 803 (26.6 %)	No *n* = 2,217 (73.4 %)		Yes *n* = 244 (8.1 %)	No *n* = 2,776 (91.9 %)	
Residence in rural areas when 1–15 years old			234.80***			28.78***
Yes	761(94.8)	1,492(67.3)		217(88.9)	2,036(73.3)	
No	42 (5.2)	725(32.7)		27(11.1)	740(26.7)	
High school located in rural areas			252.17***			21.04***
Yes	732(91.2)	1,350(60.9)		200(82.0)	1,882(67.8)	
No	71(8.8)	867(39.1)		44(18.0)	894(32.2)	
Parents current residence in rural areas			281.14***			25.11***
Yes	754(93.9)	1,386(62.5)		207(84.8)	1,933(69.6)	
No	49(6.1)	831(37.5)		37(15.2)	843(30.4)	

Note: (1) Percentage in parenthesis; (2) *** statistically significant at the 1 percent level

### Logit models

Table 5 presented summary results of the six Logit models (detailed results are listed in Additional file 3, Additional file 4, Additional file 5, Additional file 6, Additional file 7 and Additional file 8).

**Table 5:** Summary Results of Logit Model Estimations

	Logit Model 1	Logit Model 2	Logit Model 3	Logit Model 4	Logit Model 5	Logit Model 6
Dependent variable	Preference for employment in rural areas			Preference for employment in public PCPs		
Key independent variable	Residence in rural areas when 1–15 years old	High school in rural areas	Parents’ current residence in rural areas	Residence in rural areas when 1–15 years old	High school in rural areas	Parents’ current residence in rural areas
β	2.046	1.730	2.124	0.849	0.512	0.683
Robust standard error	0.181	0.148	0.171	0.245	0.198	0.213
*z*	11.260	11.670	12.450	3.460	2.580	3.210
*p*	0.000	0.000	0.000	0.001	0.010	0.001
N	3020	3020	3020	3020	3020	3020
Log pseudo likelihood	−1566.388	−1566.027	−1540.058	−806.748	−810.765	−808.347
Waldχ^2^ (22)	247.730	285.020	270.280	74.220	64.770	70.290
Prob > χ^2^	0.000	0.000	0.000	0.000	0.000	0.000
Pseudo R^2^	0.104	0.105	0.120	0.048	0.044	0.047

Medical students who preferred to work in rural areas upon graduation were more likely to be those lived in rural areas when 1–15 years old (β = 2.05, p < 0.001) (Model 1), had high school in rural areas (β = 1.73, p < 0.001) (Model 2), and had parents’ place of current residence in rural areas (β = 2.12, p < 0.001) (Model 3). Similarly, medical students who prefer to work in PCPs were more likely to be those lived in rural areas when 1–15 years old (β =0.85, p < 0.01) (Model 4), had high school in rural areas (β =0.51, p < 0.05) (Model 5), and had parents’ place of residence in rural areas (β =0.68, p < 0.01) (Model 6).

## Discussion

This study provides rich information regarding medical students' career preferences, reasons for their career choices, and the relationship between rural background with preference for rural and PCP employment. By collecting data from multiple institutions across China, this study contributes to the literature by improving the generalizability of findings.

We found that medical undergraduates mainly preferred to work in public hospitals or pursue graduate study. Students who pursue graduate study do so in public hospitals where they are exposed to employment possibilities. Therefore the percentage of public hospital preference should be more than reported in this study. Consistent with previous findings, this study found that only a minority of medical students preferred to work in rural areas and PCPs [14, 15, 24]. Reasons for not seeking employment in rural areas/PCPs were lack of opportunities for professional development and potential for job promotion, social connection with life in cities, and satisfactory reward [25–28]. Our study also found that main factors influencing medical undergraduate' career choices are career prospects, geographic location, and remuneration. By comparison, reports from developed countries on barriers to work in rural areas include poor working conditions, low job satisfaction, political problems, ethical problems, and poor security [29, 30]. Factors predisposing medical undergraduates to work in rural vs. urban areas are likely context-specific.

We found rural students are more likely to prefer to work in rural areas and in PCPs. Again, these findings correspond well to findings from relevant research in China [17, 31–33] and other countries [34–37]: rural exposure is positively associated with subsequent practice in rural areas and in PCPs. Students with rural backgrounds presumably have more real contact with rural population, and are more aware of the gap between their health needs and the access to health care. Exposure to the hardship regarding health access of underserved people in rural areas may help to establish internal empathy and balance out the appreciation of good rewards and promising career prospects. Medical students with rural background may found it easier to fit into the rural environment.

This study has several implications. First, schemes to increase the number of health professionals in rural and PCPs should start with recruiting medical applicants with rural backgrounds. However, the current admission process of medical schools, with emphasis mainly on academic performances, tends to put rural students in a disadvantaged position. For example, autonomous admission and directly admission of excellent high school students may favor financially advantaged students who are more likely to be from cities with more access to resources for academic preparation. It is worth noting that targeted enrollment of rural students has been implemented by the government to provide health professionals for township health centers in middle and western China [38]. Second, in guiding medical students’ career planning, medical schools should present a sense of duty to the needs of the population, many of whom reside in more rural settings. Third, medical schools should orient students to rural and primary care interests by more innovative strategies, such as providing rural replacement or volunteer opportunities to work in rural health services/PCPs, or emphasizing rural health, primary health care, and medical professionalism as components in formal curriculum.

This study has a few limitations. First, the randomness and representative of the data may be threatened because sampled private medical schools did not participated in the survey and the stratification sampling did not take into account some potentially important characteristics variables because they were not released or collected, such as student body demographics and origins and exposure to rural and urban medicine. Hence, the Logit models may be biased and inconsistent due to selection bias. Nevertheless, public medical schools are dominant in China. Data in 2012 demonstrate that private medical schools account only for 16.4 % of all Chinese medical schools. Therefore the influence of not including private medical schools is minor. Second, not all the factors that may play a role in affecting medical undergraduates’ preference for working in rural areas or PCPs were included into the models, such as health and income policies. As a result, the Logit model may suffer from omitted variable bias and the coefficients may be lowered. Third, the survey was modified from an international instrument that is used to understand career preferences in other settings. Some of the questions were high-inference and open to interpretation by the respondents. Fourth, the process of conducting the questionnaires at each medical school varied and the return rates were not determined. Therefore, potential bias cannot be fully analyzed.

## Conclusions

By conducting a nationwide questionnaire survey of graduating medical students, we mapped their career preferences, described reasons for the preferences, and found that rural background was associated with preferences for working in rural areas/PCPs. The study results provide insightful implications for policy-making in medical education, including student admission, student career planning, and reforms in medical education.

### Consent for publication

Not applicable.

### Available of data and materials

The dataset on which the conclusions of the manuscript rely is presented in additional supporting files.

## Additional files

Additional file 1: Graduating Medical Undergraduates’ Career Preferences by Gender (DOCX 15 kb) Additional file 2: Reasons for Preferred Career by Gender (DOCX 16 kb) Additional file 3: Results of Logit Model 1 Estimation: predicting medical undergraduates’ willingness to work in rural areas (*N*=3020) (DOCX 17 kb) Additional file 4: Results of Logit Model 2 Estimation: predicting medical undergraduates’ willingness to work in rural areas (*N*=3020) (DOCX 17 kb) Additional file 5: Results of Logit Model 3 Estimation: predicting medical undergraduates’ willingness to work in rural areas (*N*=3020) (DOCX 17 kb) Additional file 6: Results of Logit Model 4 Estimation: predicting medical undergraduates willingness’ to work in PCPs (*N*=3020) (DOCX 17 kb) Additional file 7: Results of Logit Model 5 Estimation: predicting medical undergraduates’ willingness to work in PCPs (*N*=3020) (DOCX 17 kb) Additional file 8: Results of Logit Model 6 Estimation: predicting medical undergraduates’ willingness to work in PCPs (*N*=3020) (DOCX 17 kb)

## Abbreviations

PCP: primary care provider
RN: registered nurse
MLE: maximum likelihood estimation
SD: standard deviation
