# Supplementary material for: Career preferences of graduating medical students in China: a nationwide cross-sectional study
Source: BMC Med Educ. 2016 May 6;16:136. doi: 10.1186/s12909-016-0658-5 (PMC4859951; doi:10.1186/s12909-016-0658-5)
Supplement: Additional file 1: — Graduating Medical Undergraduates’ Career Preferences by Gender (DOCX 15 kb) [file 12909_2016_658_MOESM1_ESM.docx]

**Additional file 1: Graduating Medical Undergraduates’ Career Preferences by Gender**

|  | Upon graduation | | Five years after graduation | |
| --- | --- | --- | --- | --- |
| Preferred career | Male  (n=1332) | Female  (n=1688) | Male  (n=1332) | Female  (n=1688) |
| Public hospital | 707(53.1) | 757(44.8) | 973(73.0) | 1,249(74.0) |
| Pursuing graduate study | 441(33.1) | 693(41.1) | 121(9.1) | 124(7.3) |
| Public primary care provider | 94(7.1) | 150 (8.9) | 71(5.3) | 141(8.4) |
| Private hospital/clinic | 44(3.3) | 43(2.5) | 28(2.1) | 34(2.0) |
| Academic employment | 10(0.8) | 11(0.7) | 24(1.8) | 41(2.4) |
| Going abroad | 10(0.8) | 8(0.5) | 37(2.8) | 39(2.3) |
| Self-employed | 6(0.5) | 5(0.3) | 33(2.5) | 25(1.5) |
| Medical/pharmaceutical company | 5(0.4) | 5(0.3) | 10(0.8) | 8(0.5) |
| Non-governmental organization | 3(0.2) | 5(0.3) | 7(0.5) | 4(0.2) |
| Other | 12(0.9) | 11(0.7) | 28(2.1) | 23(1.4) |
| Chi-square | 29.93*** | | 23.51*** | |

Note: (1) Percentage in parenthesis; (2) ***Statistically significant at the 1 percent level
