# Supplementary material for: Career preferences of graduating medical students in China: a nationwide cross-sectional study
Source: BMC Med Educ. 2016 May 6;16:136. doi: 10.1186/s12909-016-0658-5 (PMC4859951; doi:10.1186/s12909-016-0658-5)
Supplement: Additional file 2: — Reasons for Preferred Career by Gender (DOCX 16 kb) [file 12909_2016_658_MOESM2_ESM.docx]

**Additional file 2: Reasons for Preferred Career by Gender**

|  | 1^st^ reason | | 2^nd^ reason | | 3^rd^ reason | |
| --- | --- | --- | --- | --- | --- | --- |
|  | Male  (n=1163) | Female  (n=1543) | Male  (n=1153) | Female  (n=1532) | Male  (n=1151) | Female  (n=1527) |
| Good career prospects | 313(26.9) | 473 (30.7) | 178(15.4) | 278(18.1) | 178(15.5) | 232(15.2) |
| Living close to parents/families | 267(23.0) | 460(29.8) | 215(18.6) | 260(17.0) | 142(12.3) | 167(10.9) |
| Remuneration | 319(27.4) | 243(15.7) | 239(20.7) | 315(20.6) | 182(15.8) | 314(20.6) |
| Nice place to live | 58(5.0) | 99(6.4) | 130(11.3) | 158(10.3) | 135(11.7) | 168(11.0) |
| Return to hometown | 53(4.6) | 71(4.6) | 98(8.5) | 96(6.3) | 79(6.9) | 60(3.9) |
| Social prestige | 42(3.6) | 44(2.9) | 53(4.6) | 78(5.1) | 92(8.0) | 117(7.7) |
| Work environment | 27(2.3) | 56(3.6) | 66(5.7) | 124(8.1) | 125(10.9) | 194(12.7) |
| Opportunity for further training | 32(2.8) | 47(3.0) | 66(5.7) | 128(8.4) | 64(5.6) | 113(7.4) |
| Get away from parents | 31(2.7) | 30 (1.9) | 50(4.3) | 30(2.0) | 16(1.4) | 18(1.2) |
| Good welfare | 14(1.2) | 9(0.6) | 41(3.6) | 54(3.5) | 94(8.2) | 106(6.9) |
| Housing | 3(0.3) | 5(0.3) | 15(1.3) | 8(0.5) | 38(3.3) | 35(2.3) |
| Other | 4(0.3) | 6(0.4) | 2(0.2) | 3(0.2) | 6(0.5) | 3(0.2) |
| Chi-square | 70.46*** | | 38.02*** | | 31.73*** | |

Note: (1) Percentage in parenthesis; (2) ***Statistically significant at the 1 percent level
