# Supplementary material for: Career preferences of graduating medical students in China: a nationwide cross-sectional study
Source: BMC Med Educ. 2016 May 6;16:136. doi: 10.1186/s12909-016-0658-5 (PMC4859951; doi:10.1186/s12909-016-0658-5)
Supplement: Additional file 3: — Results of Logit Model 1 Estimation: predicting medical undergraduates’ willingness to work in rural areas (N=3020) (DOCX 17 kb) [file 12909_2016_658_MOESM3_ESM.docx]

**Additional file 3: Results of Logit Model 1 Estimation: predicting medical undergraduates’ willingness to work in rural areas (N=3020)**

| **Variables** | **β** | **Robust Std. Error** | **95% Conf. Interval** | |
| --- | --- | --- | --- | --- |
| Whether lived in rural areas when 1-15 years old | |  |  |  |
| No | — | — |  |  |
| Yes | 2.046*** | 0.182 | 1.689 | 2.401 |
| Whether “211” university or not | |  |  |  |
| No | — | — |  |  |
| Yes | -0.858*** | 0.282 | -1.411 | -0.305 |
| Location of university |  |  |  |  |
| Eastern China | — | — |  |  |
| Middle China | 0.062 | 0.097 | -0.128 | 0.251 |
| Western China | -0.913*** | 0.340 | -1.619 | -0.208 |
| Sex |  |  |  |  |
| Female | — | — |  |  |
| Male | 0.022 | 0.088 | -0.152 | 0.195 |
| Age | 0.777 | 0.914 | -1.014 | 2.568 |
| Age^2^ | -0.014 | 0.019 | -0.051 | 0.023 |
| Family income in past 5 years | -1.19e-06* | 7.99e-07 | -2.76e-06 | 3.74e-07 |
| **Father’s education** |  |  |  |  |
| Never attended school | — | — |  |  |
| Primary school | -1.020** | 0.466 | -1.933 | -0.107 |
| High school | -1.228*** | 0.458 | -2.126 | -0.331 |
| Secondary school | -1.125** | 0.482 | -2.069 | -0.181 |
| Bachelor or Diploma | -1.464*** | 0.476 | -2.397 | -0.530 |
| Master | -1.395* | 0.711 | -2.789 | -0.001 |
| Doctor | -0.991 | 1.009 | -2.969 | 0.986 |
| Other | -1.257* | 0.696 | -2.621 | 0.106 |
| **Mother’s education** |  |  |  |  |
| Never attended school | — | — |  |  |
| Primary school | -0.045 | 0.242 | -0.520 | 0.431 |
| High school | 0.061 | 0.239 | -0.407 | 0.529 |
| Secondary school | -0.063 | 0.288 | -0.628 | 0.502 |
| Bachelor or Diploma | -0.099 | 0.293 | -0.672 | 0.475 |
| Master | 0.039 | 0.696 | -1.324 | 1.403 |
| Doctor | 0.685 | 0.992 | -1.260 | 2.630 |
| Other | -0.236 | 0.738 | -1.683 | 1.210 |

* Statistically significant at the 10 percent level

**Statistically significant at the 5 percent level

***Statistically significant at the 1 percent level
