# Supplementary material for: Career preferences of graduating medical students in China: a nationwide cross-sectional study
Source: BMC Med Educ. 2016 May 6;16:136. doi: 10.1186/s12909-016-0658-5 (PMC4859951; doi:10.1186/s12909-016-0658-5)
Supplement: Additional file 4: — Results of Logit Model 2 Estimation: predicting medical undergraduates’ willingness to work in rural areas (N=3020) (DOCX 17 kb) [file 12909_2016_658_MOESM4_ESM.docx]

**Additional file 4: Results of Logit Model 2 Estimation: predicting medical undergraduates’ willingness to work in rural areas (N=3020)**

| **Variables** | **β** | **Robust Std. Error** | **95% Conf. Interval** | |
| --- | --- | --- | --- | --- |
| Whether high school locate in rural areas | | | | |
| No | — | — |  |  |
| Yes | 1.730*** | 0.148 | 1.439 | 2.020 |
| Whether “211” university or not |  |  |  |  |
| No | — | — |  |  |
| Yes | -0.916*** | 0.263 | -1.433 | -0.400 |
| Location of university |  |  |  |  |
| Eastern China | — | — |  |  |
| Middle China | 0.018 | 0.097 | -0.172 | 0.209 |
| Western China | -0.718** | 0.371 | -1.446 | 0.010 |
| Sex |  |  |  |  |
| Female | — | — |  |  |
| Male | 0.039 | 0.089 | -0.136 | 0.213 |
| Age | 0.848 | 0.949 | -1.012 | 2.708 |
| Age^2^ | -0.015 | 0.020 | -0.054 | 0.023 |
| Family income in past 5 years | -1.49e-06* | 7.90e-07 | -3.03e-06 | 6.30e-08 |
| **Father’s education** |  |  |  |  |
| Never attended school | — | — |  |  |
| Primary school | -1.116** | 0.476 | -2.049 | -0.183 |
| High school | -1.356*** | 0.466 | -2.270 | -0.442 |
| Secondary school | -1.253*** | 0.488 | -2.210 | -0.295 |
| Bachelor or Diploma | -1.582*** | 0.484 | -2.530 | -0.634 |
| Master | -1.572 | 0.665 | -2.875 | -0.269 |
| Doctor | -1.240* | 0.836 | -2.879 | 0.400 |
| Other | -1.446** | 0.698 | -2.815 | -0.078 |
| **Mother’s education** |  |  |  |  |
| Never attended school | — | — |  |  |
| Primary school | 0.014 | 0.244 | -0.465 | 0.493 |
| High school | 0.105 | 0.240 | -0.365 | 0.576 |
| Secondary school | -0.062 | 0.290 | -0.629 | 0.506 |
| Bachelor or Diploma | -0.108 | 0.292 | -0.681 | 0.465 |
| Master | 0.148 | 0.595 | -1.018 | 1.314 |
| Doctor | 0.425 | 0.809 | -1.162 | 2.011 |
| Other | -0.243 | 0.727 | -1.668 | 1.182 |

* Statistically significant at the 10 percent level

**Statistically significant at the 5 percent level

***Statistically significant at the 1 percent level
