# Supplementary material for: Career preferences of graduating medical students in China: a nationwide cross-sectional study
Source: BMC Med Educ. 2016 May 6;16:136. doi: 10.1186/s12909-016-0658-5 (PMC4859951; doi:10.1186/s12909-016-0658-5)
Supplement: Additional file 5: — Results of Logit Model 3 Estimation: predicting medical undergraduates’ willingness to work in rural areas (N=3020) (DOCX 17 kb) [file 12909_2016_658_MOESM5_ESM.docx]

**Additional file 5: Results of Logit Model 3 Estimation**: **predicting medical undergraduates’ willingness to work in rural areas (N=3020)**

| **Variables** | **β** | **Robust Std. Error** | **95% Conf. Interval** | |
| --- | --- | --- | --- | --- |
| Whether parents live in rural areas | | | | |
| No | — | — | — | — |
| Yes | 2.124*** | 0.171 | 1.790 | 2.458 |
| Whether “211” university or not |  |  |  |  |
| No | — | — | — | — |
| Yes | -0.962*** | 0.266 | -1.482 | -0.441 |
| Location of university |  |  |  |  |
| Eastern China | — | — | — | — |
| Middle China | 0.113 | 0.096 | -0.075 | 0.301 |
| Western China | -0.875** | 0.367 | -1.595 | -0.155 |
| Sex |  |  |  |  |
| Female | — | — | — | — |
| Male | 0.034 | 0.090 | -0.141 | 0.210 |
| Age | 0.636 | 0.857 | -1.043 | 2.315 |
| Age^2^ | -0.011 | 0.018 | -0.046 | 0.024 |
| Family income in past 5 years | -1.45e-06* | 7.71e-07 | -2.96e-06 | 6.11e-08 |
| **Father’s education** |  |  |  |  |
| Never attended school | — | — | — | — |
| Primary school | -1.046** | 0.467 | -1.961 | -0.130 |
| High school | -1.236*** | 0.459 | -2.136 | -0.337 |
| Secondary school | -1.109** | 0.483 | -2.057 | -0.162 |
| Bachelor or Diploma | -1.446*** | 0.477 | -2.381 | -0.510 |
| Master | -1.490** | 0.686 | -2.834 | -0.145 |
| Doctor | -0.987 | 0.960 | -2.868 | 0.894 |
| Other | -1.158* | 0.680 | -2.490 | 0.173 |
| **Mother’s education** |  |  |  |  |
| Never attended school | — | — | — | — |
| Primary school | -0.121 | 0.245 | -0.600 | 0.359 |
| High school | -0.024 | 0.239 | -0.493 | 0.445 |
| Secondary school | -0.103 | 0.291 | -0.673 | 0.467 |
| Bachelor or Diploma | -0.161 | 0.290 | -0.730 | 0.408 |
| Master | 0.265 | 0.647 | -1.003 | 1.534 |
| Doctor | 0.448 | 0.898 | -1.311 | 2.207 |
| Other | -0.045 | 0.669 | -1.357 | 1.267 |

* Statistically significant at the 10 percent level

**Statistically significant at the 5 percent level

***Statistically significant at the 1 percent level
