# Supplementary material for: Career preferences of graduating medical students in China: a nationwide cross-sectional study
Source: BMC Med Educ. 2016 May 6;16:136. doi: 10.1186/s12909-016-0658-5 (PMC4859951; doi:10.1186/s12909-016-0658-5)
Supplement: Additional file 6: — Results of Logit Model 4 Estimation: predicting medical undergraduates willingness’ to work in PCPs (N=3020) (DOCX 17 kb) [file 12909_2016_658_MOESM6_ESM.docx]

**Additional file 6: Results of Logit Model 4 Estimation: predicting medical undergraduates willingness’ to work in PCPs (N=3020)**

| **Variables** | **β** | **Robust Std. Error** | **95% Conf. Interval** | |
| --- | --- | --- | --- | --- |
| Whether lived in rural areas when 1-15 years old | | | | |
| No | — | — |  |  |
| Yes | 0.849*** | 0.245 | 0.368 | 1.330 |
| Whether “211” university or not |  |  |  |  |
| No | — | — |  |  |
| Yes | -0.450 | 0.367 | -1.169 | 0.269 |
| Location of university |  |  |  |  |
| Eastern China | — | — |  |  |
| Middle China | 0.371** | 0.163 | 0.051 | 0.691 |
| Western China | -0.187 | 0.470 | -1.108 | 0.734 |
| Sex |  |  |  |  |
| Female | — | — |  |  |
| Male | -0.301** | 0.141 | -0.576 | -0.025 |
| Age | -0.493 | 1.561 | -3.553 | 2.566 |
| Age^2^ | 0.014 | 0.032 | -0.049 | 0.078 |
| Family income in past 5 years | -3.91e-07 | 6.88e-07 | -1.74e-06 | 9.56e-07 |
| **Father’s education** |  |  |  |  |
| Never attended school | — | — |  |  |
| Primary school | -1.068* | 0.598 | -2.239 | 0.104 |
| High school | -1.170** | 0.581 | -2.309 | -0.032 |
| Secondary school | -1.555** | 0.645 | -2.819 | -0.291 |
| Bachelor or Diploma | -1.562*** | 0.621 | -2.779 | -0.345 |
| Master | -2.213** | 1.049 | -4.268 | -0.158 |
| Doctor | -2.303* | 1.452 | -5.149 | 0.544 |
| Other | -2.041* | 1.114 | -4.223 | 0.142 |
| **Mother’s education** |  |  |  |  |
| Never attended school | — | — |  |  |
| Primary school | 0.992* | 0.535 | -0.057 | 2.040 |
| High school | 1.131** | 0.530 | 0.093 | 2.169 |
| Secondary school | 1.304** | 0.594 | 0.139 | 2.469 |
| Bachelor or Diploma | 0.700 | 0.606 | -0.489 | 1.888 |
| Master | 2.500*** | 0.824 | 0.881 | 4.112 |
| Doctor | 1.523 | 1.307 | -1.038 | 4.085 |
| Other | 1.607 | 1.113 | -0.575 | 3.790 |

* Statistically significant at the 10 percent level

**Statistically significant at the 5 percent level

***Statistically significant at the 1 percent level
