# Supplementary material for: Career preferences of graduating medical students in China: a nationwide cross-sectional study
Source: BMC Med Educ. 2016 May 6;16:136. doi: 10.1186/s12909-016-0658-5 (PMC4859951; doi:10.1186/s12909-016-0658-5)
Supplement: Additional file 7: — Results of Logit Model 5 Estimation: predicting medical undergraduates’ willingness to work in PCPs (N=3020) (DOCX 17 kb) [file 12909_2016_658_MOESM7_ESM.docx]

**Additional file 7: Results of Logit Model 5 Estimation: predicting medical undergraduates’ willingness to work in PCPs (N=3020)**

| **Variables** | **β** | **Robust Std. Error** | **95% Conf. Interval** | |
| --- | --- | --- | --- | --- |
| Whether high school locate in rural areas | | | | |
| No | — | — |  |  |
| Yes | 0.512*** | 0.198 | 0.123 | 0.901 |
| Whether “211” university or not |  |  |  |  |
| No | — | — |  |  |
| Yes | -0.475 | 0.366 | -1.193 | 0.243 |
| Location of university |  |  |  |  |
| Eastern China | — | — |  |  |
| Middle China | 0.356** | 0.163 | 0.037 | 0.675 |
| Western China | -0.105 | 0.472 | -1.031 | 0.820 |
| Sex |  |  |  |  |
| Female | — | — |  |  |
| Male | -0.288** | 0.141 | -0.563 | -0.012 |
| Age | -0.537 | 1.542 | -3.559 | 2.484 |
| Age^2^ | 0.014 | 0.031 | -0.047 | 0.076 |
| Family income in past 5 years | -5.75e-07 | 7.20e-07 | -1.99e-06 | 8.37e-07 |
| **Father’s education** |  |  |  |  |
| Never attended school | — | — |  |  |
| Primary school | -1.079* | 0.596 | -2.247 | 0.089 |
| High school | -1.191** | 0.579 | -2.323 | -0.056 |
| Secondary school | -1.580*** | 0.645 | -2.844 | -0.316 |
| Bachelor or Diploma | -1.612*** | 0.619 | -2.825 | -0.398 |
| Master | -2.281** | 1.025 | -4.291 | -0.272 |
| Doctor | -2.385* | 1.420 | -5.167 | 0.397 |
| Other | -2.092* | 1.122 | -4.290 | 0.106 |
| **Mother’s education** |  |  |  |  |
| Never attended school | — | — |  |  |
| Primary school | 1.008* | 0.532 | -0.035 | 2.051 |
| High school | 1.125** | 0.527 | 0.093 | 2.157 |
| Secondary school | 1.259** | 0.593 | 0.096 | 2.422 |
| Bachelor or Diploma | 0.615 | 0.606 | -0.572 | 1.803 |
| Master | 2.398*** | 0.805 | 0.820 | 3.976 |
| Doctor | 1.286 | 1.303 | -1.268 | 3.839 |
| Other | 1.571 | 1.121 | -0.626 | 3.768 |

* Statistically significant at the 10 percent level

**Statistically significant at the 5 percent level

***Statistically significant at the 1 percent level
