# Supplementary material for: Career preferences of graduating medical students in China: a nationwide cross-sectional study
Source: BMC Med Educ. 2016 May 6;16:136. doi: 10.1186/s12909-016-0658-5 (PMC4859951; doi:10.1186/s12909-016-0658-5)
Supplement: Additional file 8: — Results of Logit Model 6 Estimation: predicting medical undergraduates’ willingness to work in PCPs (N=3020) (DOCX 17 kb) [file 12909_2016_658_MOESM8_ESM.docx]

**Additional file 8: Results of Logit Model 6 Estimation: predicting medical undergraduates’ willingness to work in PCPs (N=3020)**

| **Variables** | **β** | **Robust Std. Error** | **95% Conf. Interval** | |
| --- | --- | --- | --- | --- |
| Whether parents live in rural areas | | | | |
| No | — | — |  |  |
| Yes | 0.683*** | 0.213 | 0.266 | 1.100 |
| Whether “211” university or not |  |  |  |  |
| No | — | — |  |  |
| Yes | -0.483 | 0.365 | -1.198 | 0.232 |
| Location of university |  |  |  |  |
| Eastern China | — | — |  |  |
| Middle China | 0.386** | 0.164 | 0.065 | 0.706 |
| Western China | -0.166 | 0.470 | -1.087 | 0.755 |
| Sex |  |  |  |  |
| Female | — | — |  |  |
| Male | -0.293** | 0.141 | -0.569 | -0.018 |
| Age | -0.645 | 1.434 | -3.455 | 2.165 |
| Age^2^ | 0.017 | 0.029 | -0.041 | 0.074 |
| Family income in past 5 years | -5.50e-07 | 7.13e-07 | -1.95e-06 | 8.46e-07 |
| **Father’s education** |  |  |  |  |
| Never attended school | — | — |  |  |
| Primary school | -1.058* | 0.598 | -2.230 | 0.115 |
| High school | -1.149** | 0.581 | -2.287 | -0.010 |
| Secondary school | -1.533** | 0.646 | -2.800 | -0.267 |
| Bachelor or Diploma | -1.556*** | 0.623 | -2.777 | -0.336 |
| Master | -2.251** | 1.029 | -4.267 | -0.235 |
| Doctor | -2.328* | 1.454 | -5.177 | 0.521 |
| Other | -1.990* | 1.107 | -4.159 | 0.179 |
| **Mother’s education** |  |  |  |  |
| Never attended school | — | — |  |  |
| Primary school | 0.971* | 0.534 | -0.076 | 2.019 |
| High school | 1.092** | 0.528 | 0.057 | 2.127 |
| Secondary school | 1.279** | 0.594 | 0.115 | 2.442 |
| Bachelor or Diploma | 0.634 | 0.603 | -0.548 | 1.816 |
| Master | 2.473*** | 0.809 | 0.888 | 4.059 |
| Doctor | 1.391 | 1.362 | -1.278 | 4.060 |
| Other | 1.663* | 1.105 | -0.502 | 3.829 |

* Statistically significant at the 10 percent level

**Statistically significant at the 5 percent level

***Statistically significant at the 1 percent level
